# A third transition in science?

**DOI:** 10.1098/rsfs.2022.0063

**Published:** 2023-04-14

**Authors:** Stuart A. Kauffman, Andrea Roli

**Affiliations:** ^1^ Institute for Systems Biology, Seattle, WA, USA; ^2^ Department of Computer Science and Engineering, Università di Bologna, Campus of Cesena, Cesena, Italy; ^3^ European Centre for Living Technology, Venezia, Italy

**Keywords:** Newtonian paradigm, affordances, set theory, indefinite uses of *X*, constraint closure, emergent creativity of the biosphere

## Abstract

Since Newton, classical and quantum physics depend upon the ‘Newtonian paradigm’. The relevant variables of the system are identified. For example, we identify the position and momentum of classical particles. Laws of motion in differential form connecting the variables are formulated. An example is Newton’s three laws of motion. The boundary conditions creating the phase space of all possible values of the variables are defined. Then, given any initial condition, the differential equations of motion are integrated to yield an entailed trajectory in the prestated phase space. It is fundamental to the Newtonian paradigm that the set of possibilities that constitute the phase space is always definable and fixed ahead of time. This fails for the diachronic evolution of ever-new adaptations in any biosphere. Living cells achieve constraint closure and construct themselves. Thus, living cells, evolving via heritable variation and natural selection, adaptively construct new-in-the-universe possibilities. We can neither define nor deduce the evolving phase space: we can use no mathematics based on set theory to do so. We cannot write or solve differential equations for the diachronic evolution of ever-new adaptations in a biosphere. Evolving biospheres are outside the Newtonian paradigm. There can be no theory of everything that entails all that comes to exist. We face a third major transition in science beyond the Pythagorean dream that ‘all is number’ echoed by Newtonian physics. However, we begin to understand the emergent creativity of an evolving biosphere: emergence is not engineering.

## 1. Introduction

Three centuries after Newton we are, we believe, at a third major transition in science. We hope to make clear the evidence and need for this transition, and the unexpected vision of the creativity of our evolving biosphere. We build upon the seminal 1972 article by physics Nobel laureate P. W. Anderson, ‘More is different’ [1], recently celebrated in *Nature Physics* [2].

We may attribute the first major transition to Newton, the invention of the differential and integral calculus, and the invention of classical physics. It is no understatement that Newton taught us how to think. Call this ‘the Newtonian paradigm’ [3]: first, find the relevant variables. In physics these are often position and momentum. Write laws of motion for these relevant variables in ordinary or partial differential deterministic equation form, or stochastic variants. Define ahead of time the boundary conditions, hence the phase space of all possible values of the relevant variables such as positions and momenta of particles of the system. For any initial condition of the relevant variables, integrate the laws of motion to obtain the entailed trajectory of the system in its phase space. It is fundamental to the Newtonian paradigm that we can and must always define the fixed phase space ahead of time.

Classical physics including general relativity gave us the ‘clockwork’ universe that will unfold deterministically with a deistic god no longer able to work miracles. The same clockwork universe renders ‘chance’ merely epistemic.

The second major transition is nothing less than the reluctant discovery of the quantum of action in 1900 [4] and Heisenberg’s uncertainty relation [5,6] that demanded a transition beyond classical physics, thence the miracles of quantum mechanics and quantum field theory [7,8]. Quantum theory, however, remains safely within the Newtonian paradigm with a prestated phase space, including Fock space, hence initial and boundary conditions, and the deterministic evolution of a probability distribution via the Schrödinger wave equation. Chance becomes ontological on most interpretations of quantum mechanics [9].

The enormous power of the Newtonian paradigm can be found outside of physics. Ecology often considers a community of species linked by nonlinear dynamical equations of motion concerning the rate of reproduction of members of each species and the food web among the species. Integration of the equations in the predefined phase space of the relevant variables may exhibit limit cycles, multiple attractors and other aspects of nonlinear dynamical systems [10].

We now wish to place ecology in a wider context. Ecology deals with a predefined set of species in a community. These provide the relevant variables, hence the predefined phase space. Over evolutionary time species come and go. The set of species and their patterns of interactions themselves evolve. In the diachronic evolution of the biosphere, new adaptations emerge, existing adaptations vanish by extinction. Ecology can hope to be valid over timescales such that the species do not evolve relevant new features or lose relevant old ones. The issue we wish to raise, and the central question of this article, asks whether we can predict or deduce the new relevant adaptive variables that arise and old ones that vanish. Can we have well founded expectations? We hope to demonstrate that the answer is ‘no’.

If we cannot deduce the ever-changing phase space it will be because we will be unable to write or solve equations of motion allowing deduction of those changing phase spaces. We will be outside of the Newtonian paradigm.

Life on earth has existed for almost 4 Gyr, almost 30% of the lifetime of the universe. A failure of the Newtonian paradigm with respect to evolving life will mean that major aspects of the cosmological evolution of the universe are outside of the Newtonian paradigm.

## 2. The non-deducible diachronic evolution of the biosphere

Life started on earth about 3.7 billion years ago. The biosphere is the most complex system we know in the universe. The central new issue is that it is not possible to deduce the diachronic evolution of our or any biosphere. The evolving biosphere is a propagating construction not an entailed deduction [11–14].

The reasons seem, at first, strange [13–15]:

1. The universe is not ergodic above the level of about 500 Da [16]. The universe will not make all possible complex molecules such as proteins 200 amino acids long in vastly longer than the lifetime of the universe [13,17]. The chemical and physical properties of the different complex molecules are different, and in biology the functional properties of these tens of thousands of these different molecules in cells are also different. The universe is not ergodic because it will not make all the possible different complex molecules on timescales very much longer than the lifetime of the universe. It is true that most complex things will never ‘get to exist’.
2. Human hearts, very complex things weighing 300 g and able to function to pump blood, exist in the universe. How can that be possible? The fundamental answer for why hearts exist in the universe is that life, based on physics, arose, evolved and adapted in that evolution over time. Living things have a special organization of non-equilibrium processes. Living things are Kantian wholes where the parts exist for and by means of the whole. Humans are Kantian wholes. We exist for and by means of our parts, such as hearts pumping blood, and kidneys purifying the blood, then in the loops of Henle making and excreting urine. Because we, as Kantian wholes, propagate our offspring, our sustaining parts, hearts and kidneys are also propagated and evolve to function better. The ‘function’ of the heart is to pump blood, not jiggle water in the pericardial sac. The *function* of a part is that subset of its causal properties that sustains the whole [13].
3. We cannot hope to account for the existence in the universe of a heart that can pump blood, or the loop of Henle in the kidney that can purify urine, without appeal to the function of these organs and their adaptive diachronic evolution by Darwin’s heritable variation and natural selection. Selection is downward causation. Selection acts on the whole organism, not its evolving parts. What gets to exist in the evolving biosphere is that which was selected. The explanatory arrows point upward. The selection of the whole alters the parts.
4. In more detail, a Kantian whole has the property that the parts exist for and by means of the whole. A simple physical example is an existing nine peptide collectively autocatalytic set [13,18]. Here peptide 1 catalyses a reaction forming a second copy of peptide 2 by ligating half fragments of peptide 2 into a second copy of peptide 2. The half fragments are ‘food’ fed from an exogenous source. Similarly, peptide 2 catalyses the formation of a second copy of peptide 3. And so on around a cycle in which peptide 9 catalyses a second copy of peptide 1. The entire set of nine peptides is collectively autocatalytic. The set is a Kantian whole.
  This collectively autocatalytic physical set has these properties:
  i. It is collectively autocatalytic [18]. No molecule catalyses its own formation. Thus, this is a *Kantian whole*, the parts do exist for and by means of the whole.
  ii. The function of a part is that subset of its causal properties that sustains the whole. The function of peptide 1 is to catalyse the formation of a second copy of peptide 2. If, in doing so, the peptide jiggles the water in the Petri plate, that causal consequence is not the function of peptide 1 [13,14,17,19].
  iii. The system achieves *catalytic closure*: all reactions needing catalysis have catalysts within the same system.
  iv. The system achieves the newly recognized and powerful property of constraint closure [12]: thermodynamic work is the constrained release of energy into a few degrees of freedom [20]. These constraints constitute boundary conditions. The peptides in the nine peptide collectively autocatalytic set are each a physical boundary condition that constrains the release of chemical energy: each peptide binds the two substrates of the next peptide, thus lower activation barrier, thus chemical energy is released into a few degrees of freedom, and thermodynamic work is done to ligate the two fragments and construct the next peptide. *Critically, the set of peptides construct themselves, thus construct the very constraints on the release of energy that constitutes the work by which they construct themselves. This is constraint closure* [12–14].
  Cells literally construct themselves. The evolving biosphere constructs itself. Automobiles do not construct themselves. We construct our artefacts. Living cells constitute a new class of matter and organization of process that is a new union of thermodynamic work, catalytic closure and constraint closure [12]. In a real sense this is the long sought ‘*vital force*’, here rendered entirely non-mystical.
  Because living cells are open thermodynamic systems that construct themselves, they construct ever-new boundary conditions that thereby create new-in-the-universe phase space possibilities [13,14]. Because boundary conditions change, ever-new ‘relevant variables’ emerge and constitute the new phase space. For example, with respect to the heart, systolic blood pressure, diastolic blood pressure, cardiac blood ejection volume and blood oxygenation are among the now functionally relevant variables. How are we to account for this without adaptive evolution of ever novel functionalities?
5. Adaptations in the evolution of the biosphere are *affordances*, typically seized by heritable variation and natural selection. An example of an affordance [21] is a horizontal surface which affords you a place to sit. Affordances are, in general ‘The possible use by me of *X* to accomplish *Y*’. ‘Accomplish’ can occur without ‘mind’, but by ‘blind’ heritable variation and natural selection, as in the evolution of the heart and loop of Henle [15].
  An affordance is not an independent feature of the world [22]. An affordance is in relation to the evolving organism for whom it is an affordance to be seized or not by heritable variation and natural selection. Biological degrees of freedom are affordances, or relational opportunities available to evolving organisms.
6. Often in evolution adaptations emerge by *co-opting* the same organ for a new function. These are called Darwinian preadaptations or exaptations [23].

Typical examples of such an affordance, or new Darwinian preadaptation, seized by heritable variation and natural selection include flight feathers, which evolved earlier for functions such as thermal insulation or as bristles but were co-opted for the new function of flight [24,25], and lens crystallins originated as enzymes [26].

A wonderful example is the evolution of the swim bladder that emerged in a lineage of fish [27]. In this latter instance, the ratio of air and water in the swim bladder functions to assess neutral buoyancy in the water column. Palaeontologists believe the swim bladder arose from the lungs of lung fish. Water got into some lung, now a sac filled with a mixture of air and water, so poised to evolve into a swim bladder. This is precisely finding a new use for the same initial ‘thing’, the lung. A new function, neutral buoyancy in the water column, has emerged in the evolving biosphere.

The fundamental new issue is this: *is it possible to prestate by deducing all possible Darwinian preadaptations in the evolution of the biosphere from 3.7 billions years ago to some point in time in the distant future such as 400 Myr from now?* We now aim to show that this is *not possible*.

## 3. The insuperable limits of set theory

In the evolution of the biosphere, ever-new phase spaces with new boundary conditions and new relevant variables arise that were not prestated. Then can those now relevant variables have been prestated? The surprising answer, we hope to show, is ‘no’. We must fail because we can neither compute, predict, nor deduce ahead of time the coming into existence of new affordances and newly relevant variables seized by heritable variation and natural selection.

We must fail because we cannot use set theory or any mathematics based on it, to reliably and soundly model the evolutionary emergence of adaptations as ‘seized affordances’. The considerations are a bit unexpected and focus on the implications of biosphere evolution features for the foundations of set theory [15]. We stress that what we shall provide is not a mathematical proof but a conceptual demonstration.

An example from the tool usage context may be greatly explicative. How many ‘uses’ does a screwdriver have, alone or with other things, in London on 22 March 2025? (i) Screw in a screw, (ii) open a can of paint, (iii) wedge a door closed, (iv) scrape putty off a window, (v) as an *objet d’art*, (vi) tie to a stick and spear a fish, (vii) rent the spear to local fishermen and take 5% of the catch and (viii) lean the screwdriver against a wall, place plywood propped up by the screwdriver and use this to shelter a wet oil painting, etc.

Is the number of uses of a screwdriver alone or with other things a specific number, say 11? No. Is the number of uses infinite? How would we know? The number of uses of a screwdriver now and over the next 1000 years is ‘*indefinite*’ or perhaps ‘*unknown*’. No one in 1690 could have used a screwdriver to short an electric connection. It is essential to remark that *we cannot list all the possible uses of a screwdriver* [13] as not only can we not predict the possible future niches for the screwdriver, but the uses of a screwdriver also depends upon user’s goals and repertoire of actions [22]. The same considerations apply in general to any object, e.g. to the uses of an engine block. It can be used to build an engine, as a chassis for a tractor, as a paper weight, to crack open coconuts against its sharp corners, etc.

Perhaps we can list all the uses of a screwdriver by applying enumeration or deduction? This is not possible either. There are four mathematical ordering scales: nominal, partial order, interval, ratio. The uses of an object are merely a *nominal* scale, therefore, there is no ordering relation between these uses. Furthermore, in general a specific use of an object does not provide the basis for entailing another use. Hence, there is no *deductive relation* between the different uses of an object, e.g. it is not possible to deduce the use of an engine block to crack open coconuts from its use as a paper weight.

These arguments hold also for the emergence of adaptations as seized affordances along the diachronic evolution of the biosphere: ever-new affordances appear which are seized by evolution and shape ever-new niches and biological adaptive functions in an unprestatable way. We now show that we cannot use set theory to deduce the emergence of ever-new adaptations in the diachronic evolution of the biosphere [15].

A first axiom of set theory is the *axiom of extensionality*: ‘Two sets are identical if and only if they contain the same members’ [28]. But we cannot prove that the *un-listable* uses of a screwdriver are identical to the un-listable uses of an engine block, as we cannot prove, once and for all, the uses of object *X*. Therefore, no axiom of extensionality. Without the axiom of extensionality sets cannot be defined. Hence, no sound set theory can be formulated.

Worse, the implications also reach mathematical fields based on set theory. The axiom of choice [29], which comes into play whenever a choice function cannot be defined, cannot be applied. The axiom of choice is equivalent to ‘well ordering’ [30], but an ordering among the unordered uses of *X* cannot exist.

A consequence of this argument is the impossibility of using numbers with respect to the emergence of novel functions in the evolving biosphere. One way to define numbers uses set theory [31]. The number ‘0’ is defined as the set of all sets each of which has 0 elements. In our case this corresponds to ‘the set of all objects that have exactly 0 uses’. Well, no, this cannot be grounded on objects, agents and goals [22].

The alternative approach to numbers is via Peano’s axioms [32]. These require a null set and a successor relation. But we have no null set. More, the different uses of *X* are unordered. We have no successor relation.

Therefore, with respect to all diachronically emerging adaptations via seizing affordances, no numbers. No integers, no rational numbers, no equations such as 2 + 3 = 5. No equations, so no irrational numbers. No real line. No equations with variables. No imaginary numbers, no quaternions, no octonions. No Cartesian spaces. No vector spaces. No Hilbert spaces. No union and intersection of uses of *X* and uses of *Y*. No first order logic. No combinatorics. No topology. No manifolds. No differential equations on manifolds. Further, without an axiom of choice, we cannot integrate and take limits on the differential equations we cannot write.^1^

Our claim is of major importance. We cannot use the mathematics of set theory with respect to the emergence of new affordances, opportunities, seized by heritable variation and natural selection, or by behaving organisms acting in their worlds. This has wide implications. Our central claim that we cannot use mathematics based on set theory, ‘The world is not a theorem’, has been published elsewhere [15]. Based on this, we conclude that organisms acting in the world cannot be universal Turing machines, hence general artificial intelligence is ruled out [35]. In turn this leads us to consider the plausibility that mind is quantum and that qualia are associated with collapse of the wave function [36] as also supposed by von Neumann [37], Wigner & Margenau [38], Shimony [39] and others [40].

Can we find a formal mathematical/logical proof of our claim? We think not. Our claim is not parallel to Gödel’s famous theorem. Gödel’s work is entirely within the formal framework of mathematics. He proves that from a set of axioms there will be formally undecidable statements. In the present case we are not within a formal framework. An effort to find a formal proof of our claim would seem to require using the mathematics of set theory to formally prove that we cannot use the mathematics of set theory. Indeed, our very claim is that there is a vast world outside of what we can capture using set theory.

## 4. The third transition: we are beyond the Newtonian paradigm

These facts mean that we are, surprised or not, at the third major transition in science. If we can neither write nor solve differential equations for the diachronic evolution of adaptations in the biosphere, we are beyond the Newtonian paradigm. Recent work in the new field of biocosmology, on independent grounds, also concludes that an evolving biosphere is beyond the Newtonian paradigm [17,19].

The evolving biosphere advances into the adjacent possibles it creates, but we cannot deduce what is ‘in’ that adjacent possible. Therefore, we do not know the sample space of the process, hence can neither define a probability measure, nor define ‘random’. We truly have no well-founded expectations. This contrasts sharply with the common Kolmogorov axioms of probability [41] where the sample space must be known.

The implications are very large. If we can write and solve no equations for the diachronic evolution of our or any biosphere and our evolving universe has at least one evolving biosphere, there can be no theory of everything that entails what comes to exist in the evolving universe. The famous equation destined for the T-shirt [42], it now seems, does not exist.

This result is somewhat stunning at first, then perhaps not totally surprising. Godel’s first incompleteness theorem [43] assures us that any consistent axiomatic system as rich as arithmetic has the property that, given the axioms and the inference rules, a statement exists such that it can neither be proved nor disproved inside the system. The non-provable statement is itself generated algorithmically [44]. If this algorithmically generated statement itself is added to the initial axioms, the new set of axioms again algorithmically generates statements whose truth cannot be neither proved nor disproved.

The evolving biosphere instantiates Godel’s theorem, but far more. New adaptations, new uses of physical things such as molecules, as is true for the new uses of an engine block, cannot be deduced from the old uses. And importantly, affordances are referential degrees of freedom, not independent physical features of the world. Thus, the referential new uses cannot be deduced as a theorem from knowledge of the properties and functions of the existing molecules and other physical properties of organisms prior to the new adaptation [15]. Therefore, they are more than the analogue of merely algorithmically generated undecidable statements: They can be read as *If I get to exist in a new way for some time in the biosphere, my new existence, indeed even the very possibility of my existence, cannot be deduced from the biosphere up to the present moment*.

Our claim is further assured by recent results demonstrating that no modeller in the universe can construct a complete model of the universe [45]. Again, there can be no theory of everything.

Reluctant or not, we observe that the evolution of our or any biosphere is outside of the Newtonian paradigm. What are some implications?

1. There really can be no ‘theory of everything’ that entails all that comes to exist in the evolving universe. The dream of such a theory of everything is magnificent and has been a driving motivation for superb science for centuries. Perhaps our arguments are wrong. If so, let them be vanquished.
2. The evolution of our or any biosphere in the universe is not only entailed by no law, but seems not even mathematizable by known techniques. Perhaps we can invent new mathematics.
3. If no law entails the evolution of biospheres and that evolution cannot even be mathematized, biological evolution is radically ‘free’ to be and is vastly creative. Section 5 below hopes to find some of the unexpected reason for such ongoing creativity.
4. Most essentially, we really are at a third transition in science. If Heisenberg’s uncertainty relation demanded a transition beyond classical physics, our incapacity to use set theory to deduce the evolution of the biosphere seems to portend another major transition. The scale and meanings of this are quite unclear at present. Our universe is creative in ways we have not known. Our understanding of the world will change.

**Figure 1.**
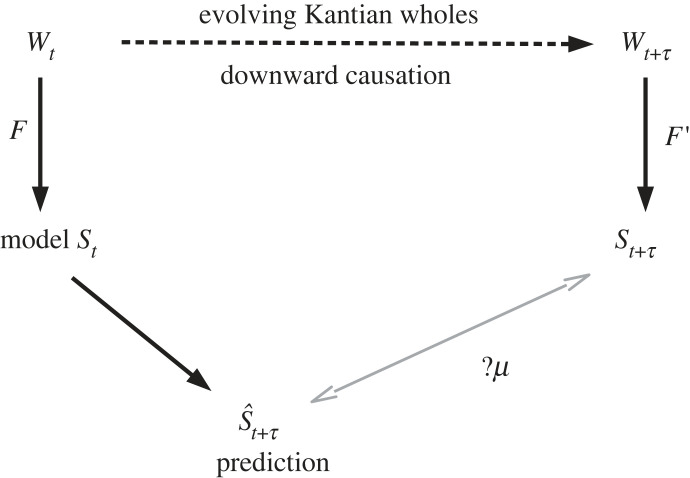
A schematic and abstract view of the main elements and relations involved in our argument. *W*_*t*_ denotes the ‘world’ at time *t*; after an interval *τ* the world changes. Biological evolution with the emergence of new Darwinian preadaptations and new relevant variables occurs. Organisms are Kantian wholes where the parts exist in the universe for and by means of the whole. Natural selection is *downward causation*. *The explanatory arrows point upward*. What comes to exist cannot be deduced from below. Reductionism fails. Selection acts on the Kantian whole and propagates its parts as they evolve new adaptations to better sustain the Kantian whole. We can use no mathematics based on set theory to deduce these new adaptive features. Thus there is no mathematical mapping based on set theory and perhaps more broadly from the biological world at time *t* to the biological world at *t* + *τ*. Therefore, the hope for a mapping *μ* that is a prediction from a model of the world at time *t* + *τ* to the actual world at *t* + *τ* does not exist. We can neither deduce the evolution of the biosphere nor estimate the errors in our predictions.

An illustration of our conceptual demonstration is provided in figure 1.

## 5. The evolution of integrated functionality: emergence is not engineering

If the evolution of life cannot be deduced and we must give up our beloved Newtonian paradigm of an entailed world, a vast new, unsuspected, world comes into view.

We achieve a new understanding of the almost miraculous emergent self-construction and emergent coherent functional organization of processes in an evolving biosphere: there is no deductive relation between the different uses of any physical thing, such as a protein in a cell that can evolve to be used to catalyse a reaction, to carry a tension load or to host a molecular motor on which it walks. Moreover, cells physically construct themselves and evolve by heritable variation and natural selection *ever seizing non-deducible new affordances*.

Therefore, each molecule and structure in evolving cells and organisms in the biosphere stands ever-available to be co-opted and selected, alone or with other things, for indefinite adaptive new uses such that myriad new adaptations and new physical things such as new proteins arise all the time. The new uses are not open to deduction from the old uses.

Yet, magically, functional integration is always maintained, even as it transforms, because the functional evolution of the parts must always be integrated into and sustain the functioning Kantian whole upon which selection acts.

Selection acting upon the whole determines what ‘gets to exist’ for some time in the non-ergodic biosphere. This is ‘downward causation’. The explanatory arrows do not point only downward [46].

The evolving biosphere really is a propagating adapting construction, not an entailed deduction. This is ‘sustained functional integrated emergence’ in evolving Kantian wholes. It is the arrival of the fitter.

*This is emergence. Emergence is not engineering*. This radical emergence of a co-evolving biosphere itself emerges only beyond the Newtonian paradigm. That we are at a third transition in science, beyond Newton’s wonderful paradigm, is not a loss, rather it is an invitation to participate in this magical emergence we have not even seen before.

We hardly begin to understand this. An evolving biosphere is a self-constructing, functionally integrated blossoming emergence. This new understanding shares common ground with the old Buddhist concept of co-dependent origination [47].

An evolving biosphere is a propagating construction, not an entailed deduction.

The evolving biosphere is ever-emergent and creative. Co-evolving fungi and bacteria in the soil create ever-new ‘possibility bubbles’ that alter the phase space of the biosphere. The third transition in science demands new tools, experiments and observations to understand how the evolving biosphere and global economy persistently create new possibilities that cannot be deduced.

A central implication of this third transition in science is that we cannot ‘command and control’ the evolving biosphere we share.

Hiding behind the equations we write, we cannot see the reality that they hide: the mystery of evolving life. We are of it, not above it.

## 6. Conclusion

The twenty-first century promises to be the century of biology. This embraces of course the explosion of biotechnology, an emergence of 21st century medicine, and ever deeper analysis of how cells and organisms that now exist ‘work’ as physical systems at molecular, cellular, organism and ecosystem levels. Here reliance on physics, chemistry, biophysics, biochemistry and molecular biology is essential. The issues are massive in complexity and import. We are in the era of systems biology.

However, we confront the third major transformation in science, following Newton and quantum mechanics, the first two transformations. We are forced beyond the wonderful Newtonian paradigm. There really is no ‘theory of everything’: The diachronic evolution of our or any biosphere is beyond entailing law and beyond any mathematics based on set theory.

There may well be 10^19^ biospheres in the universe. Evolving biospheres are immensely creative in ways beyond our knowing or stating. We live forward in face of mystery. This implies that we humans are of nature, not above nature. Rather than a loss, this is, instead, an enormous invitation. We can try to understand in new ways how our or any biosphere, our global economy and even our cultures diachronically construct themselves over billions, millions and hundreds of thousands of years of non-deducible, non-entailed, ever creative, non-ergodic emergence. We are inevitably invited to see reality anew. We are inevitably invited to live responsibly, respectfully and in wonder as we share co-creating the evolving reality of the biosphere.

## Data Availability

This article has no additional data.

## Endnote

1 Both the ϵ–*δ* formal definition of limits [33] and the one based on *infinitesimals* [34] rely on set theory.
